# Age-Related Adaptation of Bone-PDL-Tooth Complex: *Rattus-Norvegicus* as a Model System

**DOI:** 10.1371/journal.pone.0035980

**Published:** 2012-04-30

**Authors:** Narita L. Leong, Jonathan M. Hurng, Sabra I. Djomehri, Stuart A. Gansky, Mark I. Ryder, Sunita P. Ho

**Affiliations:** 1 Division of Biomaterials & Bioengineering, University of California San Francisco, San Francisco, California, United States of America; 2 Division of Oral Epidemiology & Dental Public Health, Department of Preventive and Restorative Dental Sciences, University of California San Francisco, San Francisco, California, United States of America; 3 Division of Periodontology, Department of Orofacial Sciences, University of California San Francisco, San Francisco, California, United States of America; University of Rochester, United States of America

## Abstract

Functional loads on an organ induce tissue adaptations by converting mechanical energy into chemical energy at a cell-level. The transducing capacity of cells alters physico-chemical properties of tissues, developing a positive feedback commonly recognized as the form-function relationship. In this study, organ and tissue adaptations were mapped in the bone-tooth complex by identifying and correlating biomolecular expressions to physico-chemical properties in rats from 1.5 to 15 months. However, future research using hard and soft chow over relevant age groups would decouple the function related effects from aging affects. Progressive curvature in the distal root with increased root resorption was observed using micro X-ray computed tomography. Resorption was correlated to the increased activity of multinucleated osteoclasts on the distal side of the molars until 6 months using tartrate resistant acid phosphatase (TRAP). Interestingly, mononucleated TRAP positive cells within PDL vasculature were observed in older rats. Higher levels of glycosaminoglycans were identified at PDL-bone and PDL-cementum entheses using alcian blue stain. Decreasing biochemical gradients from coronal to apical zones, specifically biomolecules that can induce osteogenic (biglycan) and fibrogenic (fibromodulin, decorin) phenotypes, and PDL-specific negative regulator of mineralization (asporin) were observed using immunohistochemistry. Heterogeneous distribution of Ca and P in alveolar bone, and relatively lower contents at the entheses, were observed using energy dispersive X-ray analysis. No correlation between age and microhardness of alveolar bone (0.7±0.1 to 0.9±0.2 GPa) and cementum (0.6±0.1 to 0.8±0.3 GPa) was observed using a microindenter. However, hardness of cementum and alveolar bone at any given age were significantly different (P<0.05). These observations should be taken into account as baseline parameters, during development (1.5 to 4 months), growth (4 to 10 months), followed by a senescent phase (10 to 15 months), from which deviations due to experimentally induced perturbations can be effectively investigated.

## Introduction

The change in morphology or form of an organ throughout development, growth, and senescent phases of an organism, is mediated by genetic and epigenetic factors [1], [2], [3], [4], [5]. The inherited genetic influences dominate morphogenesis in pre-function, which then become basal to the epigenetic factors (for e.g. functional loads) that mediate organ adaptation over a prolonged time [5]. Prolonged time alters form-function behavior due to load-related changes at an organ level and strain-related events at a cellular level.

Cellular events are identified through genetic and protein expressions, which in turn promote physico-chemical changes in tissues. Physico-chemical changes include mineral formation or resorption, changes in elemental composition, and mechanical resistance of extracellular matrices of tissues. Collectively, these processes occur in several adjoining tissues of the load bearing joint and play a key role in maintaining its functional efficiency. Hence, over prolonged time, tissues adapt to functional demands to maintain mechanical efficiency of an organ [5], [6]. However, adaptation over prolonged time also includes effects due to physiological aging of an organism. Acknowledging that aging is a science in and of itself, we present in this study the specific changes in biochemical and physico-chemical properties of the bone-tooth complex in younger, middle-aged, and older rats. This allows us to better understand the rat periodontium and its appropriateness as an animal model for various applications.

The bone-tooth organ in rats is extensively used as a model system to study the effects of various external perturbations, which include extraneous loads and disease states [7], [8]. Systematic studies [9] at the cell, tissue, and organ levels [10] continue to be performed on rats because of the limited availability of whole human bone-tooth complexes. Examples include but are not limited to periodontal diseases (gingivitis, periodontitis) [11], [12], [13], [14], [15], [16], [17] and load-mediated effects (traumatic loads using orthodontic braces [7], [18], over load using hyper-occlusion [19] and disuse using hypo-occlusion [20], which parallel skeletal bone adaptation [21]. Despite all these studies, few age-related studies exist on the bone-tooth complex in humans or other model organisms [22], [23], [24]. Selection of appropriate animal age groups is imperative, as it plays an underlying role when investigating effects of experimental variables. This is because the effect of an experimental variable could be compounded or masked by innate age-related changes (i.e. injury in younger developing patients form minimal scar tissue, in contrast to the more extensive scar tissue formation in older patients). While younger animal models may still be undergoing the complex processes of development and maturation, those in the senescent phase may already be compromised by age associated diseases [25], [26].

Age-related differences can also be translated into biochemical and physico-chemical changes, critical to accurate interpretation of experimental factors. These changes in part could be due to the varying response of the mechanosensory system and/or hormonal influences, both of which alter the mechanical efficiency of muscle, bone, and their respective interfaces [27]. There is evidence supporting the loss of functional efficiency in a joint with age due to an innate decrease in metabolic rate of cells within mineralized and soft tissues, as well as the soft-hard and hard-hard tissue interfaces [28], [29], [30], [31], [32], [33]. To understand function-related changes, time-related functional adaptation of bone was intelligently delineated as modeling, compared to the innate remodeling [34], [35], [36] of tissues. Modeling dominates in growing rats, where gains in bone formation, strength, and mass were observed due to the dominance of muscle mass in shaping bone as functional units. However, from adulthood (i.e. load-bearing organs well into function), extraneous loads and significant deviations from physiological threshold limits could affect the form or shape of load bearing functional units [37]. Age-related changes in organ biomechanics, were related to the changes in PDL mechanical properties, turnover rate, PDL-width and density [38], along with increased cementum apposition [39]. However, the common denominator for all developed periodontal tissues is functional load. As a result, the rate of change in functional loads with age can alter the rate of mechanobiological events with age [40], [41]. These mechanobiological events could manifest as biophysical changes within the load bearing bone, cementum and PDL in rats [42], [43].

Markers to identify physico-chemical changes in load bearing tissues include varying distribution and relative contents of inorganic and organic constituents, as well as their resulting mechanical properties. It is well known that across mammalian species, the inorganic constituents in various forms of apatite [44], [45], [46] and organic constituents [47], such as proteoglycans (PGs), other noncollagenous proteins, and collagen, together affect tissue and interface mechanics [24], [48]. Low and high molecular weight glycosaminoglycan (GAG) contents associated with small and large proteoglycans (PGs) were observed to decrease with age in the human periodontium [49], joint articular discs [50], bone [51], and cartilage [52]. In the rat, the amount of PGs decrease in the temporomandibular joint [53], brain [54], and skin [55], with no studies to our knowledge on the rat periodontium. A disproportionate amount of studies continue to be focused on the effects of disease and/or loading of the periodontium, but lack information on age-related changes in structure, chemical composition, and mechanical properties, all of which maintain the load bearing function of the periodontium [16], [23], [56], [57], [58], [59]. The importance of mapping changes in tissues and organs with respect to time/age addresses the fact that the transducing capacity of cells alters the load bearing nature of tissues, developing a positive feedback, commonly recognized as the form-function relationship of an organ.

This investigation provides various insights at organ and tissue levels of a bone-tooth complex in animal models (i.e. the Sprague Dawley rat) with an increase in age. Specifically, three factors were considered: 1) the ongoing basal adaptation maintaining the tooth-bone complex, 2) changes inherent to the process of aging (providing a timeline from development, growth, through senescence) and 3) identification by mapping changes in biochemical and physico-chemical properties. The innate changes in tissues and organs need to be understood so that meaningful experimental results can be efficiently and accurately extrapolated to intended clinical problems. The results of this investigation set the stage for future studies on distinguishing age-related effects from function-related effects. Function-related effects can be better understood by correlating protein expressions and tissue related physico-chemical changes modulated by diet stiffness [42].

## Results

### 2.1. Occlusal Wear and Morphology of the Root

**Figure 1:**
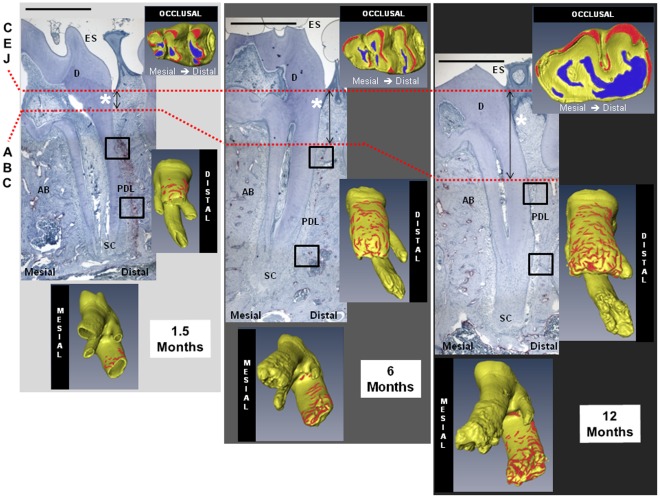
Morphological changes and anatomical locations within TRAP positive cells. Morphological changes were observed in histological sections and confirmed in three dimensional space using a MicroXCT (shown in insets comparing occlusal, distal, and mesial surfaces). The distal preference of TRAP positive cells and progressive mesial curving were observed. Anatomical structures include enamel space (ES), dentin (D), periodontal ligament (PDL), alveolar bone (AB), and secondary cementum (SC). TRAP stained micrographs at 4X magnification show first molar distal roots decreasing in osteoclast intensity and frequency with age. Representative coronal and apical TRAP positive areas in the encircled regions are enlarged in Figure 3. With age, a transition from CEJ to DGJ (demarcated by white asterisk) and increasing recession (CEJ-ABC distance represented by distance between dashed red lines) were observed. Scale bar = 1 millimeter. CEJ: cementoenamel junction; DGJ: dentin gingival junction; ABC: alveolar bone crest.

Light micrographs complemented by micro x-ray computed tomography (MicroXCT) volumetric reconstructions (Fig. 1 and Fig. S2) illustrated structural changes in molars as rats aged. Although in occlusal function by 1.5 months, complete development marked by a closed root apex was not noted until the 4 month time point. From a functional perspective, occlusal wear preceded and continued after complete root formation, which occurred within the 1.5 to 4 month window (Fig. S2). In the reconstructed tomographs, the occlusal surfaces were designated as blue for wear and red for regions of highest contact. Similarly, cratered root surfaces were designated red to represent the density of root resorption. Morphology changes with age were confirmed by an increase in the size of the mandible (Fig. S1) [60] and dentition [61], [62], but more interestingly by the mesial curving of the roots caused primarily by secondary cementum apposition (Fig. 1). Root morphology was characterized by the shape and overall root angle with respect to the occlusal plane. Roots in 1.5 month old rats were straight compared to 4 month old rats with mesial curving, and by 10 months the roots assumed a “J" shape (Fig. 1 and Fig. S2).

### 2.2. Structural Evaluation of the Bone-PDL-cementum Complex

**Figure 2:**
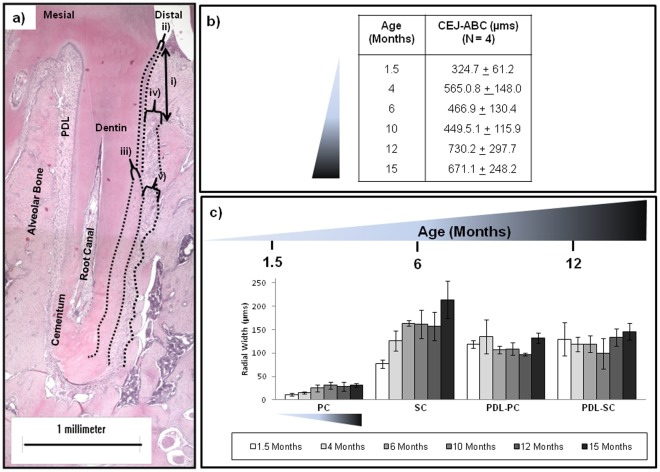
Histomorphometry as a function of age. a) Representative light micrograph of first molar distal root at 4X magnification illustrates histomorphometric parameters including i) CEJ-ABC distance, radial widths of: ii) primary cementum, iii) secondary cementum (SC), iv) PDL adjoining primary cementum (PDL-PC), and v) PDL adjoining secondary cementum (PDL-SC). Scale bar = 1 millimeter. b) The table illustrates increasing values of CEJ-ABC distance signifying recession with age. c) Plotted histomorphometric parameters as a function of age comparatively demonstrates increasing cementum and decreasing PDL trends with age.

The measured structural parameters were i) cementoenamel junction–alveolar bone crest (CEJ-ABC) distance and the radial widths of ii) primary cementum (PC), iii) secondary cementum (SC), iv) periodontal ligament space adjoining primary cementum (PDL-PC), v) periodontal ligament space adjoining secondary cementum (PDL-SC) (Fig. 2a). The CEJ-ABC distance increased with age (Figs. 1, 2b), a measure of decreasing bone height. Another observation was the loss of CEJ (Fig. 1) around 6 months in the form of a butt joint and the appearance of a gap CEJ [15], [63] with further increase in age. Consistent with other observations [60], [61], [62], increasing cementum apposition, measured as radial width, particularly in SC was noted in rats from 14 to 500 days, (Fig. 2c). While PC spanned the majority of the coronal length in younger age groups, increasing SC deposition spanned the root length with age. Overall PDL-space demonstrated a gradual decrease after 1.5 months and an increase around 10 and 12 months (Fig. 2c). Significant differences (ANOVA P<0.05) in coronal PDL-PC width were observed until 10 months, while apical PDL-SC width differed significantly at 1.5 and 15 months (Fig. 2c). Cement lines in bone of younger rats were observed more on the distal side, but became increasingly prominent on the mesial side with age (data not shown).

### 2.3. TRAP Histochemistry for Clastic Activity of Bone and Cementum

**Figure 3:**
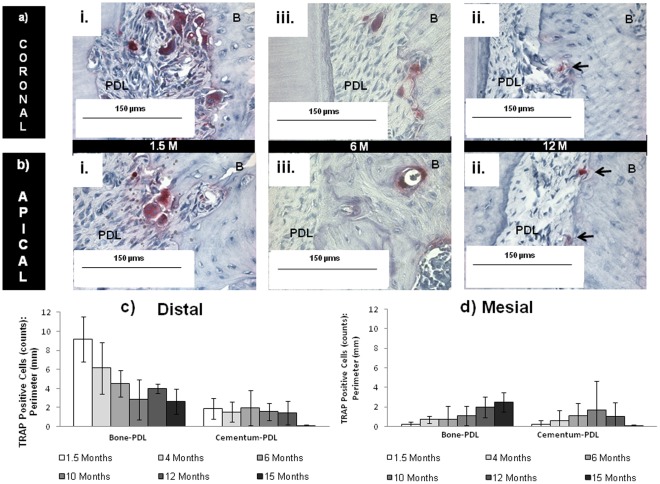
Regions of osteoclastic activity with age. Enlarged at 40X from encircled areas in Figure 1, TRAP localization is demonstrated across all age groups in a) coronal and b) apical regions. In young rats, resorption activity is represented by robust multinucleated pits (red staining) surrounded by several dark purple nuclei. In older rats mononucleated pits predominated with decreased intensity of red stain. Scale bar = 150 µm. Results show osteoclastic activity declined with increasing age in c) distal regions and d) increased in mesial regions.

Bone, PDL and cementum within the complex form a continuum through interfaces, although often considered as discrete tissues. Resorption at the PDL-bone and PDL-cementum attachment sites was identified in all age groups with tartrate resistant acid phosphatase (TRAP) staining for positive mineral resorption cells on soft-hard interfaces observed on all molars (Figs. 1, 3). Quantification of TRAP positive cells confirmed distal preference. Mesial and distal sides were comparable in active resorption only in older 12–15 month age groups (Figs. 3c, 3d). Based on TRAP staining, PDL-cementum resorption was less frequent relative to that of PDL-bone resorption, but was observed in younger 1.5, 4, and 6 month old rats. Overall TRAP positive activity declined in staining intensity and number with age (Figs. 3a, 3b, Figs. S1, S2). Young rats exhibited multinucleated TRAP positive osteoclasts, whereas old rats predominantly contained mononucleated TRAP positive cells. Mononucleated TRAP positive cells were observed at the soft-hard tissue interfaces as well as endosteal and marrow spaces in interradicular and apical bone, including the PDL-space (Figs. 1, indicated by black arrows in 3a, 3b, Fig. S3). TRAP positive clastic activity was complemented by resorption pits observed in tomographies of the adjoining root surfaces (Fig. 1, Fig. S2). The complementary information supports preference of distal apical root resorption and diminishing region specificity with age.

### 2.4. Alcian Blue Staining for High and Low Molecular Weight Proteoglycans

**Figure 4:**
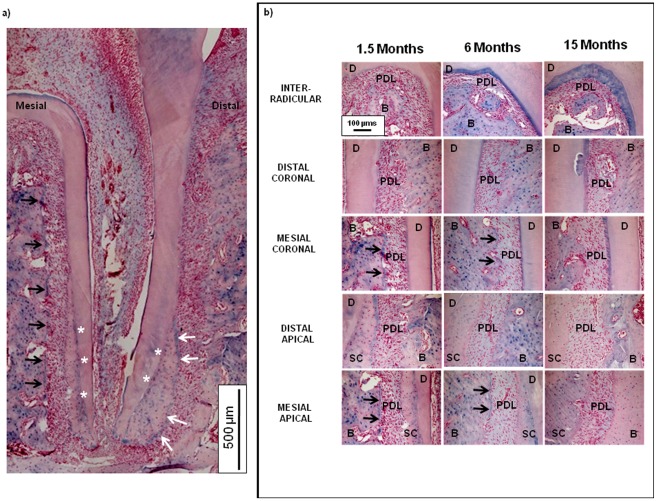
Localization of sulfated GAGs using alcian blue stain. a) Light micrographs at 10X of alcian blue with nuclear fast red counterstain indicates more sulfated GAGs on the mesial aspect as indicated by black arrows (shown here in a 1.5 month old rat), as well as around osteocytes in the alveolar bone, precementum (white arrows) and cementum dentin junction (white asterisks). We also observed staining to occur in dentin. Scale bars = 500 µm. b) Corresponding age-related changes at interradicular, coronal-apical, and mesial-distal entheses regions showed decreased alcian blue staining and lost mesial specificity with age (black arrows). Scale bars = 100 µm.

Localization of alcian blue (Fig. 4) opposes TRAP positive staining (Figs. 1 and 3) in the younger 1.5 month age group. Alcian blue was consistently observed on the PDL-bone enthesis of the mesial and interradicular surfaces of the roots in younger rats (Fig. 4a, black arrows) with minimal stain at the distal PDL-bone complex. With age, alcian blue was predominantly found in the PDL-cementum entheses of the mesial, coronal, and interradicular root surfaces (Fig. 4b). Regardless of age, alcian blue staining was consistently found around the osteocytes in the alveolar bone, precementum (Fig. 4a, white arrows) and cementum dentin junction (Fig. 4a, asterisks). Increasing intensity of alcian blue stain in the younger groups (growth phase), decreasing intensity in the older groups (senescent phase), and no coronal-apical patterns were observed. Staining was also observed in dentin (mantle dentin, predentin), closest to the occlusal surface (not shown).

### 2.5. Immunostaining for SLRPs

**Figure 5:**
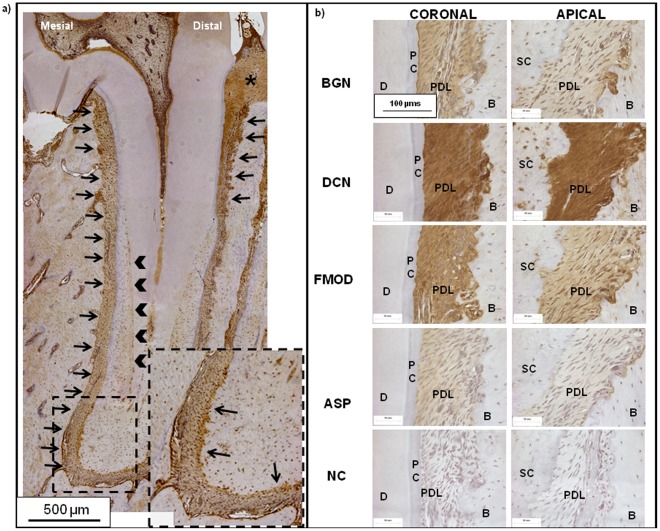
Immunohistochemical staining for identification of SLRPs. a) Light micrograph at 10X using immunohistochemical methods shows representative localization of SLRPs (biglycan expression in a 6 month old rat) counterstained with hematoxylin. Black arrows indicate localization at mesial PDL-bone enthesis spanning the root length as well as the coronal predominance. In addition localization was also observed in precementum layers (black arrow heads, in Fig. 5a) and at the CDJ (black arrow in inset, Fig. 5a), predentin, and the transseptal fibers (asterisk, Fig. 5a) between any two molars. Scale bars = 500 µm. b) Serially sectioned 60X light micrographs show immunohistochemical localization of SLRPs: biglycan (BGN), decorin (DCN), fibromodulin (FMOD), asporin (ASP), and negative controls (NC). Localization indicates more intense staining in coronal regions relative to apical regions across all age groups. All micrographs indicate regions where PDL meets primary bone (B) and primary cementum (PC) or secondary cementum (SC), with adjacent dentin (D). All Scale bars = 100 µm.

Immunohistochemistry located biglycan (BGN), decorin (DCN), fibromodulin (FMOD), and asporin (ASPN) coronally, apically, and at the enthesis regions of PDL-cementum and PDL-bone (Fig. 5) with increasing age. Biglycan, decorin (Class I SLRPs), and fibromodulin (a Class II SLRP) were consistently located at these soft-hard interfaces, endosteal spaces, and vasculature independent of age. The exception was asporin, a leucine rich protein with a unique aspartate-rich N-terminus that is not a proteoglycan (PG) [64], and was confirmed to be specific to PDL in rats. A localization gradient in all SLRPs was observed with higher concentrations coronally relative to apical regions in both distal and mesial sides of all specimens in all age groups (Fig. 5b). Immunostaining for SLRPs other than asporin was also observed at the precementum layers (black arrow heads, inset in Fig. 5a), CDJ (black arrows in inset, Fig. 5a), predentin, and the transseptal fibers (asterisk, Fig. 5a) between any two molars. Shown in Figure 5a is immunostaining for BGN, which was observed to localize at the mesial side of the root across all age groups.

### 2.6. Scanning Electron Microscope

**Figure 6:**
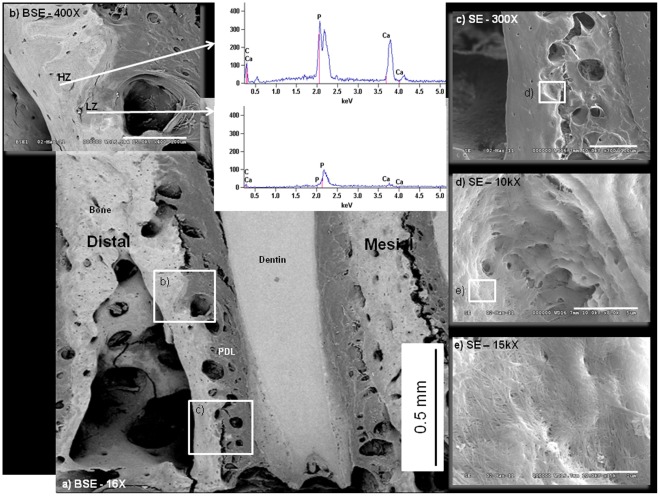
Elemental mapping using BSE and EDS. a) SEM in BSE mode indicated distribution of high and low Z elements of the periodontium. Scale Bar = 0.5 mm. b) Using SEM-BSE greatest heterogeneity is observed in the different layers of bone owing to different chemical compositions of bone during its turnover (i.e. remodeling or modeling). Scale Bar = 100 µm. Representative point shoot EDS demonstrate varying counts of Ca and P (Kα1, Kα2, and Kβ1 lines) within adjacent regions. SE mode indicates at length scales c) the enthesis region of PDL-bone with scale bar = 100 µm, d) the site of a resorption pit, which indicate the digested layers of bone with scale bar = 5 µm, and e) the exposed collagen fibers that help compose a mineralized matrix with scale bar = 4 µm.

Back scattered electron mapping [65] on rat molars across age illustrated increased distribution of lighter and darker gray regions indicating heterogeneity owing to distributions of higher and lower atomic number (Z) elements in the alveolar bone of older rats (Figs. 6a, 6b). Observations were confirmed by point analyses using EDS elemental mapping of relative counts of calcium and phosphate, in which all age groups consistently demonstrated calcium and phosphate Kα1, Kα2, and Kβ1 lines in lighter areas (higher average Z; HZ in Figs. 6a and 6b) compared to corresponding lines in the darker areas (lower average Z; LZ in Figs. 6a and 6b) areas. EDS analyses demonstrated higher counts of Ca and P in more mature and lighter layers of bone compared to newly laid bone near resorbed sites at PDL-bone enthesis (Fig. 6b). Standard SE images illustrated resorption sites with the presence of collagen fibers inserting on the distal side of bone at the PDL-bone enthesis (Figs. 6c, 6d, 6e) and into dentin at the tooth side.

### 2.7. Microhardness (see Appendix S1)

The Box-Tidwell non-linearity test [66] and linear regression test for association of mineral tissue hardness (cementum and bone; dependent variables) with age (independent variable) conducted via Statistics Online Computational Resource (SOCR) (http://www.socr.ucla.edu/) did not show a significant non-linear relationship between age and hardness of secondary cementum or alveolar bone (both P = 0.94). This implies that neither the hardness of bone nor that of cementum was non-linearly related to age of the organism. The linear regression test did not show a significant linear relationship between microhardness and age (both P>0.46). Similarly the ANCOVA test of differences in material hardness (secondary cementum and alveolar bone) and age concluded no significant relationship (P of age = 0.194). However, a significant relationship between material and hardness (P of material = 0.007) at a specific age group was demonstrated, with point estimate of the difference of hardness = −0.105 and 95% confidence of −0.163 to −0.046. However this relationship was not modified by age (P of age*material = 0.880).

## Discussion

This is the first cross-sectional study to map function related changes in structure, (bio)chemical composition, and mechanical properties, from which differential changes due to experimentally induced perturbations can be optimally addressed. The results of this study are discussed giving due consideration to Wolff’s Law, continuous occlusal wear, and natural distal drift of rat molars [7], [8], [60], [62], [67], [68], [69]. Based on our observations, we speculate that each tissue’s functional response is unique and the synchronization of individual physical and chemical properties could explain functional maintenance of the bone-PDL-cementum complex at an organ level.

### Physical Changes at an Organ and Tissue Levels: MicroXCT and Histomorphometry

Macroscale changes due to overall growth of a root and adjacent bone adaptation within the bone-tooth fibrous joint (Fig. 1, Fig. S2) were illustrated using MicroXCT. These changes can be related to functional loads, which act as stimuli to cells within various tissues and interfaces. Upon loading, molars pivot about the interradicular bone, which is considered as the natural fulcrum [70]. Age-related changes in muscle efficiency and occlusion [71] result in different chewing forces [72] over time. As a result, functional loads alone can cause tension, compression, shear and related mechanical strains within the fibrous matrix between the bone and tooth. The effect of matrix-related strains [21], [73], [74], [75], and fluid flow (blood and interstitial fluid) [86], [87] can be traced to changes in gene expressions of stem cells within the marrow space of the alveolar bone and blood vessels of the PDL. A good example is the origin of RANKL, a molecule involved in osteoclastogenesis, which can be traced from the vascularized PDL to bone marrow space containing stromal and hematopoietic stem cells [21], [73]. As a result, histomorphometric changes of load-bearing tissues with time included increased radial width of cementum and increased bone recession (measured as the vertical distance between the CEJ and the ABC) (Fig. 2c). Observed trends are reflective of bone “carving," which would conceivably be due to functional adaptation [76], [77] and in turn could alter tooth attachment (Fig. 2b) to bone (Fig. 6) to preserve optimal function space. Although age-related increases in cementum width and decreases in PDL-space were observed (Fig. 2), the combined radial widths of SC and PDL-SC remained a constant in younger rats 1.5 to 6 months and in older rats 12 to 15 months (ANOVA P≤0.05). The functional role of SC and PDL in load adaptation [15] provide the basis for considering the coupled PDL-cementum thickness in cooperative maintenance of the dynamic complex. Localized remodeling of bone is thought to dominate and allows for the uniform size increases in the growing periodontium between 1.5 to 4 months. When in function, modeling becomes more prevalent with complete secondary cementum formation and apposition from 4 to 15 months, simultaneously carving bone equivalent to tooth morphology as a result of functional stimuli.

Changes in function can be identified as compromised PDL attachment and closing of endosteal spaces in rats 12 to 15 months of age (Figs. 1, 3, 4). Generally, cement lines in bone increased, exhibiting a dominant lamellar-like pattern around endosteal and marrow spaces with age. The observed patterns can be associated with remodeling related events, which were identified in endocortical and marrow regions in aging rats [78]. Based on narrowing endosteal spaces in aging rats, it is plausible that changes in PDL-width, bone and root surface are related to changes in blood flow, supply of nutrients, and chemotactic factors responsible for turnover of tissues. Shaping of the alveolar socket or modeling at endocortical and marrow regions, compromised turnover rate of the PDL in aging rats [78], [79], including changes in PDL-width, suggests the deterioration of PDL’s mechanical integrity. However, it should be noted that observed increases in bone recession could be due to several factors. These include natural hair and food impaction, which stimulate local inflammation and bone recession. Conversely, heightened reception to hormones during the period of social maturation at 6 and 10 months (Fig. 2b) have been associated with a bone protective effect due to anabolic increases in rats at 4 months, with stabilization by 5–9 months [80].

Bone “carving" and resulting form of the socket, is functionally stimulated by clastic and blastic activities within the bony socket in which the tooth is suspended. Function related stimulation promotes bone modeling in addition to the physiological remodeling that is innate to most tissues within an organ [21], [36], [81]. This phenomena can be captured in the lifetime process of distal tooth-drift [67], and when combined with function, imposes a continuous tension-based mesial complex and a compression-based distal complex. Pre-strained functional complexes cause mineral formation on the mesial side in tension and mineral resorption on the distal side in compression. The mesial apposition of mineral, marked as stratified growth, and distal resorption of mineral, identified by its pitted border, continue to be illustrated through fluorochrome and TRAP analyses [23]. As previously delineated, our TRAP analysis embraces this logic appropriately, and can be seen as distal osteoclastic preference in regions of compression (Figs. 1, 3c, 3d, Fig. S2). However, with increasing age, no particular preference was observed with the distal resorption compared to that of the mesial formation (Figs. 3c, 3d). This observation can be argued with the predominance of mononucleated TRAP positive cells within PDL-space (Fig. 3a, 3b), indicative of a decline in osteoclastic maturation with age [82], [83]. The αvβ3 integrin and vitronectin receptor, commonly expressed in platelets, has been implicated in successful fusion of mononucleated osteoclastic precursors [84]. Additionally, increased formation of advanced glycation end-products with age interferes with the integrin binding RGD sequence [85]. This, in addition to the narrowing of blood vessels and endosteal spaces, could explain the compromised mechanotransduction and the subsequent lack of cell-matrix interactions to promote maturation of a mononucleated osteoclast to a multinucleated osteoclast with age. Additionally, it is important to note that the varying numbers of mononucleated osteoclasts within the PDL support the theory of their hematopoetic origin and possibly reflect upon their declining presence and ability to migrate from the vasculature within PDL and bone with age.

### Biochemical Changes at the Soft-hard Interface Levels in a Pre-strained Complex

It is proposed that formation and resorption related effects identified at the mesial and distal complexes are prompted by cellular activity equivalent to tension- and compression-strain gradients within respective complexes and at the soft-hard tissue interfaces. Mineral formation in a bone-tooth complex can be first identified at the osteoid and the precementum layers. However, based on our results, formation does not only occur at the interfaces. Mineral resorption related events also occur, but more specifically at the distal side complex. To better understand events responsible for formation and resorption of bone and cementum the localization of load resisting and dampening biochemical molecules, namely the higher molecular weight PGs using alcian blue, and lower molecular weights (i.e. SLRPs) using immunohistochemistry, were targeted. It is known that PGs in general are also responsible for water retention, fibrillogenesis, and the maintenance of tissue architecture and strain relieving properties (i.e. the scaffold upon which cell migration and mineralization occurs) [86]. Specifically, we identified PGs at the mesial and distal sites within the dynamic bone-tooth complex.

Biomolecules are also chemotactic agents that facilitate durotaxis, the stiffness dependent migration of cells [87]. The location and intensity of these biomolecules during development is dictated by genetic inheritance, but is predominantly governed by function when in growth. Regardless, in both development and growth, PGs are responsible for maintaining the organic matrix upon which mineral formation or resorption can occur. Moreover, this implies that in compressed regions, mineral resorbing molecules would be more dominant (Fig. 1), in contrast to tension regions that would contain more mineral forming molecules (Fig. 4a). In this study, biochemical events were mapped at a macroscale using alcian blue (Fig. 4) and more locally by identifying SLRP localization (Fig. 5). Interestingly, alcian blue stained sulfated GAGs were identified in newly formed bone sites (i.e. mesial trailing edge compared to the distal leading edge), as confirmed by others [88]. We also observed increasing gradients of sulfated GAGs on the mesial sides of PDL-bone and PDL-cementum sites, as indicated by alcian blue stains at these respective regions (Fig. 4). We observed GAG localization in regions of decreased osteoclastic activity by correlating alcian blue stain with TRAP positive stain (Figs. 3 and 4). With age, an increase in GAG localization was observed at PDL-PC, while decreases at PDL-SC and PDL-bone occurred until distal-mesial regions became comparable (Fig. 4). We hypothesize that these localizations can be related to increased tensile strains in the coronal portion of the bone-PDL-cementum complex and at the attachment sites. Increased strains can be attributed to changes in organic to inorganic ratios as a result of function related active mineral formation and resorption in bone and cementum with age [89]. Higher levels of tenascin and fibronectin, including other SLRPs noted in our study [89], [90], [91], were also identified at the attachment sites. Asporin was observed only within PDL, marking its innate nonmineralizing phenotype in maintaining the functional space necessary for adequate range of tooth motion within the bony socket. From these results it is conceivable, that should functional loads vary as a result of therapy (an alteration of compression and tension sites within the bone-tooth complex via orthodontics braces) [92], the generation of grossly altered biomolecules with mineralizing phenotypes (ankylosis) and mineral resorbing phenotypes are possible [93], [94], [95], [96]. An analog in orthopedics is the age associated loss of cartilage and joint mobility under osteoarthritic conditions that are associated with decreases in asporin [97] and other proteoglycans. Other analogs include distraction osteogenesis, in which bone formation is stimulated through unidirectional forces on excised bone. These observations suggest that the synergistic nature of PGs on tension and compression-based matrices permit mineral formation or resorption related events due to function and is not limited solely to heredity. Additionally, these biochemical events could occur concomitantly, as seen by coronal-apical gradients and specifically at the attachment sites of PDL-bone and PDL-cementum, resulting in mineralization fronts as osteoid or precementum layers at the soft-hard tissue borders.

### Physical and Chemical Changes using SEM-BSE-EDS

Newly formed layers of bone (i.e. mineralizing osteoid and pre-cementum) contained lower counts of calcium (Ca) and phosphorus (P) compared to the older layers of bone [98] (Fig. 6). Formation of mineral on and at the strained attachment sites is a complex and systematic process, some of which can be marked by speciation of phosphate groups. This is because the phosphate (PO_4_^3−^) to pyrophosphate (P_2_O_7_^4−^) ionic ratio increases as physiological aging progresses [99], [100], [101]. Modeling and/or remodeling related biomineralization events can also be mapped using SEM in backscattered electron [65] mode, along with electron dispersion X-ray spectroscopy (EDS). In this study we used complementary techniques of BSE and EDS to identify higher and lower Z elements within the dynamic tissue, bone, and less dynamic, cementum (Fig. 6) [102]. Lower Ca and P in the new bone compared to older bone at sites of modeling (Fig. 5) were observed. Despite the observed heterogeneity in bone regardless of age, it is important to note that differences in younger groups may be more related to inherited genetic factors (i.e. 1.5 months – which is closer still to development), and this most often is marked by the hypertrophic growth of cells indicated by higher metabolic activity [103].

Beyond the ongoing development in the 1.5 month age group, the increased heterogeneity observed in bone from 4 to 15 months could be due to functional loads. Moreover, growth related distal drift is dependent on functional loads (a decrease in rate of distal drift identified by lack of functional loads) [20], [68], [104], [105]. As a result, we observed an increase in the number of “patchy areas" in bone, which could be due to increased differences in Ca and P contents (Figs. 6a, 6b) as a function of modeling and remodeling related events. This in turn could be necessary to maintain the functional integrity of bone (Fig. 6b) with age. At the bone-tooth organ level, we observed stratified bone formation parallel to the root length on the tension based mesial complex with lower Z (newer bone). Compression based distal complex, identified as the “leading" edge, was observed as higher Z content (older bone) and contained several resorption pits (Fig. 6c). Interestingly, PDL was attached to bone at these resorption sites, demonstrating no loss of attachment (Figs. 6d, 6e).

### Hardness Changes Using Microindentation

**Figure 7:**
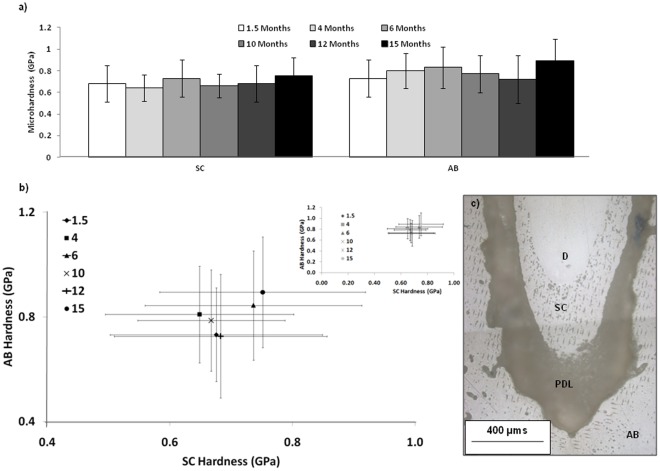
Hardness values for secondary cementum (SC) and alveolar bone (B) with age. a) Histogram shows average calculated Knoop hardness and standard deviation (GPa) values for SC and B. Values demonstrated no significant differences in both SC and B microhardness with age. b) Inset demonstrates original values plotted with reference to the origin. The main plot zooms into the clustered data set to demonstrate that the discrete points showed no relationship between SC and B with progression of age. c) Typical microindentation distribution and pattern conducted under dry conditions is shown at 10X.

The observed heterogeneity primarily in bone can be correlated to varying contents of Ca, P, and possibly other elements at certain regions, and stages of mineralization, as seen in SEM-BSE-EDS (Fig. 6). Microindentation was used to estimate hardness values representative of the tissue’s bulk properties. Several hierarchical levels of organization and substructures, from crystal formation to crystal alignment within and around the fibril, to the packaging of fibrils into a fiber [45], can affect mechanical hardness. Interestingly, mechanical testing concluded no correlation between age and microhardness of AB and SC, but microhardness of AB was significantly different from SC (see Appendix S1). Based on these results, it is conceivable that the varying chemical disparities within the patches, number of patches, and the size of patches could provide similar hardness with age. As a result, hardness (H_k_) values do not demonstrate the aging of mineralized tissues (Fig. 7) in rats. Without discriminating between new and mature bone, we pooled in the new and old bone hardness values and sampled alveolar bone as one material. Hence, it is likely that the effect of adaptation with age was not seen in hardness measurements. An engineering analogy is that different phases in a metal joined by grain boundaries could contribute to the overall functional integrity of the metal, but each phase has its own characteristic property. In the case of biological materials that are more organic based, microindentation, when conducted under dry (nonphysiological) conditions, could mask some of the innate energy absorbing characteristics of hygroscopic, organic components (i.e. GAGs, collagen) of mineralized tissues. Hence, the observed trends could be true and that only the relative differences between bone and cementum should be considered. For the same reason, a more accurate approximation can be made between AB and SC under wet (hydrated, physiological) conditions.

In summary, our results suggest that age-related physiological adaptations in organs should be taken into account, such that their use as a model can be streamlined and the effects of the intended experimental factors are accurately investigated. Specifically, we suggest that hard tissue modeling related studies in rats should not exceed 6 months. With increase in age, the adaptation in each tissue, whether they arise from evolutionarily inherent adaptations, physiological changes due to growth, or natural environmental insults, are accounted for throughout the organ system as a whole. Although each tissue’s functional response is unique owing to its characteristic cellular and acellular components, the periodontium inherently adapts as a continuum in a synchronized fashion to maintain function. Despite nature’s efficient design of the organ complex and its ability to continually withstand perturbations and functionally perform, the adaptive trend of its component tissues will be critical for future success in biomimetics. However, function-related effects should be decoupled from the effects of aging in future studies by modulating diet stiffness.

## Materials and Methods

### Ethics Statement

All animal housing, care, euthanasia, and tissue transfer protocols in this study were approved and complied with the guidelines of the Institutional Animal Care and Use Committee (IACUC) of the University of California, San Francisco and the National Institute of Health based on the IACUC Approval Number: AN083692-02.

The functional dentition of rats (1.5 months old, male, Sprague Dawley) [67] were taken as our starting time point, from which differential changes in properties will be discussed to illustrate adaptation. Experiments were performed on male Sprague-Dawley rats from six age groups: 1.5, 4, 6, 10, 12, and 15 months. Histomorphometry using hematoxylin and eosin (H&E) stained sections, alcian blue staining for sulfated glycosaminoglycans (GAGs), immunohistochemistry for small leucine rich proteins (SLRPs), and tartrate-resistant acid phosphatase (TRAP) [106] for osteoclast resorption was performed with n = 4 rats each from 1.5 to 15 month age groups using serial sections. The corresponding halves of the mandibles were used for microindentation studies. Another set of hemimandibles from all age groups (n = 3) were used for scanning electron microscopy (SEM), energy dispersive X-ray spectroscopy (EDS), and micro X-ray computed tomography (MicroXCT) analyses.

### 4.1. MicroXCT and Image Processing

Left hemimandibles from each age group were fixed in 4% paraformaldehyde (PFA) (Sigma-Aldrich, catalog P6148, St. Louis, MO), stored and imaged in 70% ethanol. Each specimen was scanned (MicroXCT-200, Xradia Inc., Pleasanton, CA) at 4X, 50 KVp, 140 µA, 2100 projections, binning 2, 7 second exposure time, and an angle sweep from –95° to 95°. At this magnification, isotropic voxel sizes were 5.5 µm with an image size of 1012 × 1012 µm. Each tomography was reconstructed using the TXM reconstructor software (Version 7.0.2817, Xradia Inc., Pleasanton, CA) to obtain DICOM image files for 3D image analysis using Visage Imaging Amira 5.3.3 (Visage Imaging, San Diego, California). Using an identical brightness and contrast threshold, root surfaces were constructed using the SurfaceGen module in Amira 5.3.3 (Visage Imaging, San Diego, CA).

### 4.2. Specimen Preparation for Histology

Hemimandibles were stored in 10% neutral buffered formalin for 4 days followed by end-stage decalcification using Cal-EX II decalcifying formalin solution (Fischer Scientific, Fair Lawn, NJ). The specimens were dehydrated with increasing concentrations of Flex 80%, 95%, and 100% alcohol (Richard-Allan Scientific, Kalamazoo, MI) before embedding in paraffin (Tissue Prep-II, Fisher Scientific, Fair Lawn, NJ) [107]. The paraffin blocks were sectioned on a rotary microtome (Reichert-Jung Biocut, Vienna, Austria) using disposable steel blades (TBFTM Inc., Shur/SharpTM, Fisher Scientific, Fair Lawn, NJ), deparaffinized with xylene, followed by rehydration with decreasing concentrations of 100%, 95%, and 80% Flex alcohols, and then stained with Hematoxylin Gill 3X (Fisher Scientific, Kalamazoo, MI) and Eosin Y Solution (Fisher Scientific, Kalamazoo, MI). For alcian blue and TRAP staining, a slight variation in specimen preparation involved tissue fixation with 4% PFA shaken overnight at room temperature and decalcified for 14 days with 0.5 M EDTA at pH 8.0. The distal root of the first mandibular molar in each rat was evaluated (see supplemental information).

### 4.3. Histomorphometry

Sagittal sections containing the maximum root length illustrating the cementoenamel junction and the pulp through the apical foramen were used for histomorphometry. Light microscopy (BX 51, Olympus America Inc., San Diego, CA), as shown in H&E stained sections of the cementum, PDL-space, and bone height of the first molar distal root complex were measured using Image-Pro Plus v6.0 data acquisition software (Media Cybernetics, Inc., Bethesda, MD). Image-Pro Plus v6.0′s magnification/zoom function was utilized to accurately measure the mentioned parameters on the distal side. 20 measurements were taken for parameters along the coronal length of the root (i.e. primary cementum (PC) and PDL-PC), 20 measurements for apical parameters (i.e. secondary cementum (SC) and PDL-SC), and one measurement for the distance between the cementoenamel junction (CEJ) and alveolar bone crest (ABC), as an indicator of bone recession [77], [108], [109], [110]. Thus, a total of 30 cementum-width and PDL-width respectively, and 1 CEJ-ABC measurements were made around the first molar along the coronal-apical length of the distal root of each specimen. Average measurements in coronal-apical regions of respective tissues for each distal root in each rat were determined. The resulting average values were used to test statistical significance among rat age groups using ANOVA with 95% confidence intervals (P≤0.05). Average values and standard deviations representative of each regional tissue for each age group were plotted.

### 4.4. TRAP Histochemistry for Osteoclasts

Deparaffinized serial sections were used for staining TRAP [111]. In brief, the method included treating the rehydrated specimens with 0.2 M acetate buffer, a solution of 0.2 M sodium acetate (Sigma Aldrich, catalog 52889, St. Louis, MO) and sodium tartrate dibasic dehydrate (Sigma Aldrich, catalog T6521, St. Louis, MO). After 20 minute incubation at room temperature, napthol AS-MX phosphate (Sigma, catalog N4875, St. Louis, MO) and fast red TR salt 1,5-napthalenedisulfonate salt (Sigma, F6760, St. Louis, MO) were added, followed by incubation at 37°C for 1 hour, with close monitoring under the microscope after the first half hour for bright red staining of osteoclastic activity. The stained sections were washed in deionized water and counterstained with Gill 3X hematoxylin (Fisher Scientific, Kalamazoo, MI) for subsequent examination under a light microscope. Distal and mesial side osteoclasts were manually counted along a measured PDL-bone and PDL-cementum perimeter using Image-Pro Plus v6.0 data acquisition software and its magnification/zoom function (Media Cybernetics, Inc., Bethesda, MD). Ratios of osteoclast count to perimeter per mesial-distal location [112], PDL-bone and PDL-cementum interfaces, were calculated across all age groups. These ratios were tested for statistical significance among age groups using ANOVA with 95% confidence intervals (P≤0.05). Additionally, average ratios and their standard deviations representative of regional osteoclastic density for each age group were plotted.

### 4.5. Alcian Blue Histochemistry for Sulfated GAGs

Deparrafinized serial sections were rehydrated in distilled water and stained in 1% alcian blue in 0.1 N hydrochloric acid at pH 1.0 for 30 minutes and then briefly rinsed in 0.1 N hydrochloric acid. Counterstaining with nuclear-fast red solution for 5 minutes was performed, followed by washing in distilled water. Dehydration with two changes each of 95% and >99% (absolute) ethyl alcohol, then deparaffinization in xylene was done before mounting the specimens. As a cationic copper containing dye, alcian blue has been widely used to stain the polyanionic sulfated GAGs at pH 1.0 [113].

### 4.6. Immunohistochemistry for SLRPs

Immunohistochemical localization of different SLRPs, specifically biglycan (BGN), decorin (DCN), fibromodulin (FMOD), and asporin (ASPN) within tissues and at the PDL-bone and PDL-cementum attachment sites (entheses) was performed. Followed by the same method for conventional histology, after rehydration of the deparaffinized sections, endogenous peroxidases were deactivated with 80% methanol and 0.6% H_2_O_2_. Further digestion for antigen retrieval was performed using a solution of 1 M Tris pH 7.4, 3 M sodium acetate pH 5.2, chondroitinase ABC (Seikagaku Biobusiness, catalog 100332) in 0.1% BSA, keratinase (Seikagaku Biobusiness, catalog 100810) in 0.1% BSA and ddH_2_O in a humidified chamber at 37°C for 60 minutes. Sections were then blocked by incubation with 1% BSA and 1.5% rabbit serum in phosphate-buffered saline at 25°C for 30 minutes. Polyclonal antibodies kindly provided by Dr. Larry Fisher (NIDCR, National Institutes of Health) were added at a dilution of 1∶50, 1∶100, 1∶100, and 1∶20 for BGN, DCN, FMOD, and ASPN, respectively, in phosphate-buffered saline, and incubated overnight at 4°C. After washing, sections were incubated with Sigma mouse anti-rabbit IgG-HRP (catalog A2074, St. Louis, MO) at a dilution of 1∶100 and then incubated at room temperature for 30 min. The sections were washed again and visualized with 3,3′-diaminobenzidine tetrachloride solution (Sigma, catalog D3939, St. Louis, MO) for immunoreaction sites. Sections were counterstained with Hematoxylin Gill 3x (Fisher Scientific, Kalamazoo, MI). Negative controls by substitution of only the primary antibody with unimmunized rabbit serum in blocking solution, while keeping other experimental steps identical, were also used for analysis.

### 4.7. SEM, BSE and EDS for Morphology, Distribution of Higher and Lower Atomic Weight Elements, and Overall Chemical Composition

Scanning electron microscopy (SEM) (Hitachi S-4300SE/N, Hitachi America) was performed to evaluate structural and chemical compositional differences at a tissue level near PDL-bone and PDL-cementum attachment sites, and in bone. Following microindentation, three samples each from the six study groups mounted in Buehler Epoxycure Resin (Buehler Ltd., Lake Bluff, IL) with surfaces polished to 1 µm using a diamond polishing suspension were coated with ∼25Å thickness gold. Imaging was performed using SEM operating at 10 keV for secondary electron (SE), 10 keV for back-scattered electron, and 15 keV for electron dispersive spectroscopy (EDS) modes. SE was used for evaluation of structure of the bone-tooth complex, while BSE was used for comparing regional variations in atomic number (Z). Areas of lower and higher Z were further explored by point analysis using EDS to compare zones of lower and higher Ca and P contents. Elemental mapping was performed predominantly measuring the Kα1, Kα2, and Kβ1 lines of calcium (orbital energy 3691.68, 3688.09, and 4012.7 eV, respectively) and phosphorus (orbital energy 2013.7, 2012.7, and 2139.1 eV, respectively) [114].

### 4.8. Mechanical Testing

Prior to indentation, hemimandibles (n = 4 per 6 age groups) were embedded in epoxy (Buehler, Lake Bluff, IL) and polished using carbide grit sizes 1200 and 2400 (Buehler Ltd., Lake Bluff, IL) followed by fine polishing using diamond suspension slurries in descending order from 6, 3, 1, to 0.25 µm (Buehler Ltd., Lake Bluff, IL). Microindentation was performed under dry conditions with a microindenter with a Knoop diamond tip (Buehler Ltd., Lake Bluff, IL) using a load of 10 gf, and the long diagonal of each Knoop indent was immediately measured with Image-Pro data-acquisition software (Media Cybernetics, Inc., Bethesda, MD) using a light microscope. The microhardness (H_K_) was calculated with the following equation: H_K_ = 0.014229P/D^2^ where P is the normal load in Newtons (N) and D (mm) the length of the long diagonal. Multiple rows of radially oriented indents were performed in secondary cementum and alveolar bone with approximately 3–5 indents per row. The distance between any two indents was chosen as per ASTM standards [115]. The mechanical properties of the first molar distal root from all groups were analyzed by first averaging the Knoop hardness values individually of bone and SC per rat. The resulting average values from each rat were used as representative hardness values for each rat within a group. Alveolar bone and SC of the first molar complex in each hemimandible were treated as separate tissues and as such, an average from all the indents of each tissue within each rat was evaluated. The resulting average values for each rat were used to test for statistical significant associations between age and hardness by performing a Box-Tidwell non-linearity test and linear regression test [66]. Additionally, the mean of the average hardness of bone and secondary cementum (representative hardness of bone and cementum in any given age group) was calculated and used to test for significant associations adjusting for age via analysis of covariance (ANCOVA) and simple regression analysis [116].

## Supporting Information

Figure S1 **Gross physical changes in hemimandible and molars with age:** TOP: Changes in the growing rat molars from younger rats at 1.5, 4, and 6 months. BOTTOM: Changes in rat molars taken from older rats at 10 and 12 months. Both top and bottom rows have insets illustrating respective hemimandibles. Alignment with centimeter ruler shows in the molar view observable increases in occlusal wear and decreases in bone height with age. In the hemimandible inset view, widening of the first molar crown appears to cease at 0.3 cm at 4 months, while mandibles appear to cease at approximately 3.75 cm in length, around 6 months of age.
(TIF)

Figure S2 **Mesial curving, occlusal wear, and resorption pits with age:** MicroXCT reconstructed roots of 1.5, 6, and 12 month first mandibular molars are shown at various buccal, angled, lingual, and occlusal views. Mesial curving becomes prominent at 6 months, as seen in the buccal and angled buccal views. Occlusal wearing is significantly observed with age, especially in the 12 month old molar, as seen in the occlusal view. Buccal and lingual views best demonstrate discrepancies between mesial and distal roots in the first molar.
(TIF)

Figure S3 **Osteoclastic activity with age:** Enlarged at 40X from encircled areas in Figure 1, TRAP localization across all age groups in respective coronal and apical regions is shown. Resorption activity appears to be localized in multinucleated pits as indicated by the red stain surrounding several dark purple nuclei. Osteoclastic activity and multinucleated cells declined with increasing age regardless of region. Scale bar = 150 µm.
(TIF)

Appendix S1 **Functional load on rat molars; Distal root of 1st molar; Box-Tidwell Regression Test.**
(DOCX)

## References

[1] Robert L, Labat-Robert J (2000). Aging of connective tissues: from genetic to epigenetic mechanisms.. Biogerontology.

[2] Fraga MF, Ballestar E, Paz MF, Ropero S, Setien F (2005). Epigenetic differences arise during the lifetime of monozygotic twins.. Proc Natl Acad Sci U S A.

[3] Wang JH, Thampatty BP, Lin JS, Im HJ (2007). Mechanoregulation of gene expression in fibroblasts.. Gene.

[4] Hayflick L (2007). Biological aging is no longer an unsolved problem.. Ann N Y Acad Sci.

[5] Carter DR, Beaupré GS (2001). Skeletal function and form : mechanobiology of skeletal development, aging, and regeneration..

[6] Brinckmann P, Frobin W, Leivseth G (2002). Musculoskeletal biomechanics..

[7] Ren Y, Maltha JC, Kuijpers-Jagtman AM (2004). The rat as a model for orthodontic tooth movement–a critical review and a proposed solution.. Eur J Orthod.

[8] Klausen B (1991). Microbiological and immunological aspects of experimental periodontal disease in rats: a review article.. J Periodontol.

[9] Struillou X, Boutigny H, Soueidan A, Layrolle P (2010). Experimental animal models in periodontology: a review.. Open Dent J.

[10] Shimono M, Ishikawa T, Ishikawa H, Matsuzaki H, Hashimoto S (2003). Regulatory mechanisms of periodontal regeneration.. Microsc Res Tech.

[11] Keles GG, Acikgoz G, Ayas B, Sakallioglu E, Firatli E (2005). Determination of systemically & locally induced periodontal defects in rats.. Indian J Med Res.

[12] Ramamurthy NS, Xu JW, Bird J, Baxter A, Bhogal R (2002). Inhibition of alveolar bone loss by matrix metalloproteinase inhibitors in experimental periodontal disease.. J Periodontal Res.

[13] Achong R, Nishimura I, Ramachandran H, Howell TH, Fiorellini JP (2003). Membrane type (MT) 1-matrix metalloproteinase (MMP) and MMP-2 expression in ligature-induced periodontitis in the rat.. J Periodontol.

[14] Kuhr A, Popa-Wagner A, Schmoll H, Schwahn C, Kocher T (2004). Observations on experimental marginal periodontitis in rats.. J Periodontal Res.

[15] Nanci A (2007). Ten Cate’s oral histology : development, structure, and function..

[16] Klausen B, Sfintescu C, Evans RT (1991). Asymmetry in periodontal bone loss of gnotobiotic Sprague-Dawley rats.. Arch Oral Biol.

[17] Ijuhin N (1988). Light and electron microscopic studies of experimentally-induced pathologic changes in the rat periodontal tissue.. Adv Dent Res.

[18] King GJ, Keeling SD, McCoy EA, Ward TH (1991). Measuring dental drift and orthodontic tooth movement in response to various initial forces in adult rats.. Am J Orthod Dentofacial Orthop.

[19] Goto KT, Kajiya H, Nemoto T, Tsutsumi T, Tsuzuki T (2011). Hyperocclusion stimulates osteoclastogenesis via CCL2 expression.. J Dent Res.

[20] Fujita T, Montet X, Tanne K, Kiliaridis S (2009). Supraposition of unopposed molars in young and adult rats.. Arch Oral Biol.

[21] Robling AG, Castillo AB, Turner CH (2006). Biomechanical and molecular regulation of bone remodeling.. Annu Rev Biomed Eng.

[22] Wang L, Banu J, McMahan CA, Kalu DN (2001). Male rodent model of age-related bone loss in men.. Bone.

[23] Misawa-Kageyama Y, Kageyama T, Moriyama K, Kurihara S, Yagasaki H (2007). Histomorphometric study on the effects of age on orthodontic tooth movement and alveolar bone turnover in rats.. Eur J Oral Sci.

[24] Hall RC, Embery G (1997). The use of immunohistochemistry in understanding the structure and function of the extracellular matrix of dental tissues.. Adv Dent Res.

[25] Buffenstein R, Edrey, H Y, Larsen, L P (2008). Animal Models in Aging Research A Critical Examination..

[26] Baer MJ, Bosma JF, Ackerman JL (1983). The postnatal development of the rat skull..

[27] Sievanen H (2005). Hormonal influences on the muscle-bone feedback system: a perspective.. J Musculoskelet Neuronal Interact.

[28] Roholl PJ, Blauw E, Zurcher C, Dormans JA, Theuns HM (1994). Evidence for a diminished maturation of preosteoblasts into osteoblasts during aging in rats: an ultrastructural analysis.. J Bone Miner Res.

[29] Hayflick L, Moorhead PS (1961). The serial cultivation of human diploid cell strains.. Exp Cell Res.

[30] Barros EM, Rodrigues CJ, Rodrigues NR, Oliveira RP, Barros TE (2002). Aging of the elastic and collagen fibers in the human cervical interspinous ligaments.. Spine J.

[31] Pereira Junior FJ, Lundh H, Westesson PL (1996). Age-related changes of the retrodiscal tissues in the temporomandibular joint.. J Oral Maxillofac Surg 54: 55–61; discussion.

[32] Klingsberg J, Butcher EO (1960). Comparative histology of age changes in oral tissues of rat, hamster, and monkey.. J Dent Res.

[33] Baumhammers A, Stallard RE, Zander HA (1965). Remodeling of alveolar bone.. J Periodontol.

[34] Frost HM (1964). Bone biodynamics..

[35] Frost HM (1990). Skeletal structural adaptations to mechanical usage (SATMU): 1. Redefining Wolff’s law: the bone modeling problem.. Anat Rec.

[36] Roberts WE, Roberts JA, Epker BN, Burr DB, Hartsfield JK (2006). Remodeling of Mineralized Tissues, Part I: The Frost Legacy.. Seminars In Orthodontics.

[37] Frost HM (1997). On our age-related bone loss: insights from a new paradigm.. J Bone Miner Res.

[38] Komatsu K, Kanazashi M, Shimada A, Shibata T, Viidik A (2004). Effects of age on the stress-strain and stress-relaxation properties of the rat molar periodontal ligament.. Arch Oral Biol.

[39] Louridis O, Bazopoulou-Kyrkanidou E, Demetriou N (1972). Age effect upon cementum width of albino rat: a histometric study.. J Periodontol.

[40] Cao JJ, Kurimoto P, Boudignon B, Rosen C, Lima F (2007). Aging impairs IGF-I receptor activation and induces skeletal resistance to IGF-I.. J Bone Miner Res.

[41] Donahue SW, Jacobs CR, Donahue HJ (2001). Flow-induced calcium oscillations in rat osteoblasts are age, loading frequency, and shear stress dependent.. American Journal of Physiology Cell Physiology.

[42] Niver EL, Leong N, Greene J, Curtis D, Ryder MI (2011). Reduced functional loads alter the physical characteristics of the bone-periodontal ligament-cementum complex..

[43] Kingsmill VJ, Boyde A, Davis GR, Howell PG, Rawlinson SC (2010). Changes in bone mineral and matrix in response to a soft diet.. J Dent Res.

[44] Posner AS, Harper RA, Muller SA, Menczel J (1965). Age changes in the crystal chemistry of bone apatite.. Ann N Y Acad Sci.

[45] Weiner S, Wagner HD (1998). The Material Bone: Structure-Mechanical Function Relations.. Annu Rev Mater Sci.

[46] Mann S (2001). Biomineralization : principles and concepts in bioinorganic materials chemistry..

[47] Bailey AJ (2001). Molecular mechanisms of ageing in connective tissues.. Mech Ageing Dev.

[48] Ho SP, Yu B, Yun W, Marshall GW, Ryder MI (2009). Structure, chemical composition and mechanical properties of human and rat cementum and its interface with root dentin.. Acta Biomater.

[49] Mariotti A (1993). The extracellular matrix of the periodontium: dynamic and interactive tissues.. Periodontol 2000.

[50] Melrose J, Fuller ES, Roughley PJ, Smith MM, Kerr B (2008). Fragmentation of decorin, biglycan, lumican and keratocan is elevated in degenerate human meniscus, knee and hip articular cartilages compared with age-matched macroscopically normal and control tissues.. Arthritis Res Ther.

[51] Grzesik WJ, Frazier CR, Shapiro JR, Sponseller PD, Robey PG (2002). Age-related changes in human bone proteoglycan structure. Impact of osteogenesis imperfecta.. J Biol Chem.

[52] Roughley PJ (2006). The structure and function of cartilage proteoglycans.. Eur Cell Mater.

[53] Okazaki J, Kamada A, Higuchi Y, Kanabayashi T, Sakaki T (1996). Age changes in the rat temporomandibular joint articular disc: a biochemical study on glycosaminoglycan content.. J Oral Rehabil.

[54] Jenkins HG, Bachelard HS (1988). Developmental and age-related changes in rat brain glycosaminoglycans.. J Neurochem.

[55] Ito Y, Takeuchi J, Yamamoto K, Hashizume Y, Sato T (2001). Age differences in immunohistochemical localizations of large proteoglycan, PG-M/versican, and small proteoglycan, decorin, in the dermis of rats.. Experimental Animals.

[56] Meikle MC (2006). The tissue, cellular, and molecular regulation of orthodontic tooth movement: 100 years after Carl Sandstedt.. Eur J Orthod.

[57] Shimizu N, Yamaguchi M, Uesu K, Goseki T, Abiko Y (2000). Stimulation of prostaglandin E2 and interleukin-1beta production from old rat periodontal ligament cells subjected to mechanical stress.. J Gerontol A Biol Sci Med Sci.

[58] Bridges T, King G, Mohammed A (1988). The effect of age on tooth movement and mineral density in the alveolar tissues of the rat.. Am J Orthod Dentofacial Orthop.

[59] Ren Y, Maltha JC, Liem RS, Stokroos I, Kuijpers-Jagtman AM (2008). Age-dependent external root resorption during tooth movement in rats.. Acta Odontol Scand.

[60] Spence JM (1940). Method of Studying the Skull Development of the Living Rat by Serial Cephalometric Roentgenograms.. The Angle Orthodontist.

[61] Hoffman MMaS, I (1940). Quantitative Studies in the Development of the Rat Molar..

[62] Applebaum E (1947). Development of rat molar crowns and jaws.. J Dent Res.

[63] Ho SP, Senkyrikova P, Marshall GW, Yun W, Wang Y (2009). Structure, chemical composition and mechanical properties of coronal cementum in human deciduous molars.. Dent Mater.

[64] Royce PM, Steinmann BU (2002). Connective tissue and it heritable disorders : molecular, genetic, and medical aspects..

[65] Afonso L, Bandaru H, Rathod A, Badheka A, Ali Kizilbash M (2011). Prevalence, determinants, and clinical significance of cardiac troponin-I elevation in individuals admitted for a hypertensive emergency.. J Clin Hypertens (Greenwich).

[66] Box GEP, Tidwell PW (1962). Transformation of the Independent Variables..

[67] Schneider BJ, Meyer J (1965). Experimental Studies on the Interrelations of Condylar Growth and Alveolar Bone Formation.. Angle Orthod.

[68] Sicher H, Weinmann JP (1944). Bone Growth and Physiologic Tooth Movement.. American journal of orthodontics and oral surgery.

[69] Enlow DH (1967). Morphogenic interpretation of cephalometric data.. J Dent Res.

[70] Chattah NL-T, Shahar R, Weiner S (2009). Design Strategy of Minipig Molars Using Electronic Speckle Pattern Interferometry: Comparison of Deformation under Load between the Tooth-Mandible Complex and the Isolated Tooth.. Advanced Materials.

[71] Nishijima K, Kuwahara S, Ohno T, Miyaishi O, Ito Y (2009). Occlusal tooth wear in male F344/N rats with aging.. Arch Gerontol Geriatr.

[72] Bani D, Bani T, Bergamini M (1999). Morphologic and biochemical changes of the masseter muscles induced by occlusal wear: studies in a rat model.. J Dent Res.

[73] Robling AG, Turner CH (2009). Mechanical signaling for bone modeling and remodeling.. Crit Rev Eukaryot Gene Expr.

[74] Ingber DE (2006). Cellular mechanotransduction: putting all the pieces together again.. FASEB J.

[75] Ingber DE (2004). The mechanochemical basis of cell and tissue regulation.. Mech Chem Biosyst.

[76] Oehmke MJ, Schramm CR, Knolle E, Frickey N, Bernhart T (2004). Age-dependent changes of the periodontal ligament in rats.. Microsc Res Tech.

[77] Klausen B, Evans RT, Sfintescu C (1989). Two complementary methods of assessing periodontal bone level in rats.. Scand J Dent Res.

[78] Frost HM, Jee WS (1992). On the rat model of human osteopenias and osteoporoses.. Bone Miner.

[79] Saffar JL, Lasfargues JJ, Cherruau M (1997). Alveolar bone and the alveolar process: the socket that is never stable.. Periodontol 2000.

[80] Ghanadian R, Lewis JG, Chisholm GD (1975). Serum testosterone and dihydrotestosterone changes with age in rat.. Steroids.

[81] Frost HM (1987). Bone “mass" and the “mechanostat": a proposal.. Anat Rec.

[82] Arai F, Miyamoto T, Ohneda O, Inada T, Sudo T (1999). Commitment and differentiation of osteoclast precursor cells by the sequential expression of c-Fms and receptor activator of nuclear factor kappaB (RANK) receptors.. Journal of Experimental Medicine.

[83] Boyle WJ, Simonet WS, Lacey DL (2003). Osteoclast differentiation and activation.. Nature.

[84] Boissy P, Machuca I, Pfaff M, Ficheux D, Jurdic P (1998). Aggregation of mononucleated precursors triggers cell surface expression of alphavbeta3 integrin, essential to formation of osteoclast-like multinucleated cells.. J Cell Sci 111 ( Pt.

[85] Paul RG, Bailey AJ (1999). The effect of advanced glycation end-product formation upon cell-matrix interactions.. International Journal of Biochemistry and Cell Biology.

[86] Iozzo RV, Murdoch AD (1996). Proteoglycans of the extracellular environment: clues from the gene and protein side offer novel perspectives in molecular diversity and function.. FASEB J.

[87] Carter SB (1967). Haptotaxis and the mechanism of cell motility.. Nature.

[88] Takagi M, Maeno M, Kagami A, Takahashi Y, Otsuka K (1991). Biochemical and immunocytochemical characterization of mineral binding proteoglycans in rat bone.. J Histochem Cytochem.

[89] Qian L, Todo M, Morita Y, Matsushita Y, Koyano K (2009). Deformation analysis of the periodontium considering the viscoelasticity of the periodontal ligament.. Dental Materials.

[90] Lukinmaa PL, Mackie EJ, Thesleff I (1991). Immunohistochemical localization of the matrix glycoproteins–tenascin and the ED-sequence-containing form of cellular fibronectin–in human permanent teeth and periodontal ligament.. J Dent Res.

[91] McCulloch CA, Lekic P, McKee MD (2000). Role of physical forces in regulating the form and function of the periodontal ligament.. Periodontol 2000.

[92] Krishnan V, Davidovitch Z (2006). Cellular, molecular, and tissue-level reactions to orthodontic force.. Am J Orthod Dentofacial Orthop 129: 469.

[93] Santra M, Mann DM, Mercer EW, Skorski T, Calabretta B (1997). Ectopic expression of decorin protein core causes a generalized growth suppression in neoplastic cells of various histogenetic origin and requires endogenous p21, an inhibitor of cyclin-dependent kinases.. J Clin Invest.

[94] Hakkinen L, Strassburger S, Kahari VM, Scott PG, Eichstetter I (2000). A role for decorin in the structural organization of periodontal ligament.. Lab Invest.

[95] Nakamura Y, Tanaka T, Noda K, Shimpo S, Oikawa T (2003). Calcification of degenerating tissues in the periodontal ligament during tooth movement.. J Periodontal Res.

[96] Nakamura Y, Noda K, Shimoda S, Oikawa T, Arai C (2008). Time-lapse observation of rat periodontal ligament during function and tooth movement, using microcomputed tomography.. Eur J Orthod.

[97] Yamada S, Tomoeda M, Ozawa Y, Yoneda S, Terashima Y (2007). PLAP-1/asporin, a novel negative regulator of periodontal ligament mineralization.. J Biol Chem.

[98] Goldberg M, Septier D, Lecolle S, Chardin H, Quintana MA (1995). Dental mineralization.. International Journal of Developmental Biology.

[99] Groeneveld MC, Everts V, Beertsen W (1995). Alkaline phosphatase activity in the periodontal ligament and gingiva of the rat molar: its relation to cementum formation.. J Dent Res.

[100] Foster BL, Nagatomo KJ, Bamashmous SO, Tompkins KA, Fong H (2011). The Progressive Ankylosis Protein Regulates Cementum Apposition and Extracellular Matrix Composition..

[101] Whyte MP (1994). Hypophosphatasia and the role of alkaline phosphatase in skeletal mineralization.. Endocrine Reviews.

[102] Foster BL, Popowics TE, Fong HK, Somerman MJ (2007). Advances in defining regulators of cementum development and periodontal regeneration.. Current Topics in Developmental Biology.

[103] Hayflick L (1970). Aging under glass.. Exp Gerontol.

[104] Luan X, Diekwisch TG (2007). Vienna-Chicago: the cultural transformation of the model system of the un-opposed molar.. Bioessays.

[105] Holliday S, Schneider B, Galang MT, Fukui T, Yamane A (2005). Bones, teeth, and genes: a genomic homage to Harry Sicher’s “Axial Movement of Teeth".. World J Orthod.

[106] Weiner JP, Tucker AM, Collins AM, Fakhraei H, Lieberman R (1998). The development of a risk-adjusted capitation payment system: the Maryland Medicaid model.. J Ambul Care Manage.

[107] Ho SP, Kurylo MP, Fong TK, Lee SS, Wagner HD (2010). The biomechanical characteristics of the bone-periodontal ligament-cementum complex.. Biomaterials.

[108] Guggenheim B, Schroeder HE (1974). Reactions in the periodontium to continuous antigenic stimulation in sensitized gnotobiotic rats.. Infect Immun.

[109] Crawford JM, Taubman MA, Smith DJ (1978). The natural history of periodontal bone loss in germfree and gnotobiotic rats infected with periodontopathic microorganisms.. J Periodontal Res.

[110] Arai K, Tanaka S, Yamamoto-Sawamura T, Sone K, Miyaishi O (2005). Aging changes in the periodontal bone of F344/N rat.. Archives of Gerontology and Geriatrics.

[111] Erlebacher A, Derynck R (1996). Increased expression of TGF-beta 2 in osteoblasts results in an osteoporosis-like phenotype.. J Cell Biol.

[112] Sawyer A, Lott P, Titrud J, McDonald J (2003). Quantification of tartrate resistant acid phosphatase distribution in mouse tibiae using image analysis.. Biotechnic and Histochemistry.

[113] Pedlar J (1979). Histochemistry of glycosaminoglycans in the skin and oral mucosa of the rat.. Arch Oral Biol.

[114] Thompson A, Kirz, J, Attwood DT, Gullikson EM, Howells MR, Energy USDo (2009). Center for X-Ray Optics and Advanced Light Source X-ray Data Booklet.. Third ed.

[115] ASTM E384 - Standard Test Method for Knoop and Vickers Hardness of Materials.. West Conshohocken, PA: American Standard for Testing Materials International.

[116] Edwards AL (1979). Multiple regression and the analysis of variance and covariance..

