# Supplementary figures and images for: Age-Related Adaptation of Bone-PDL-Tooth Complex: Rattus-Norvegicus as a Model System
Source: PLoS One. 2012 Apr 30;7(4):e35980. doi: 10.1371/journal.pone.0035980 (PMC3340399; doi:10.1371/journal.pone.0035980)

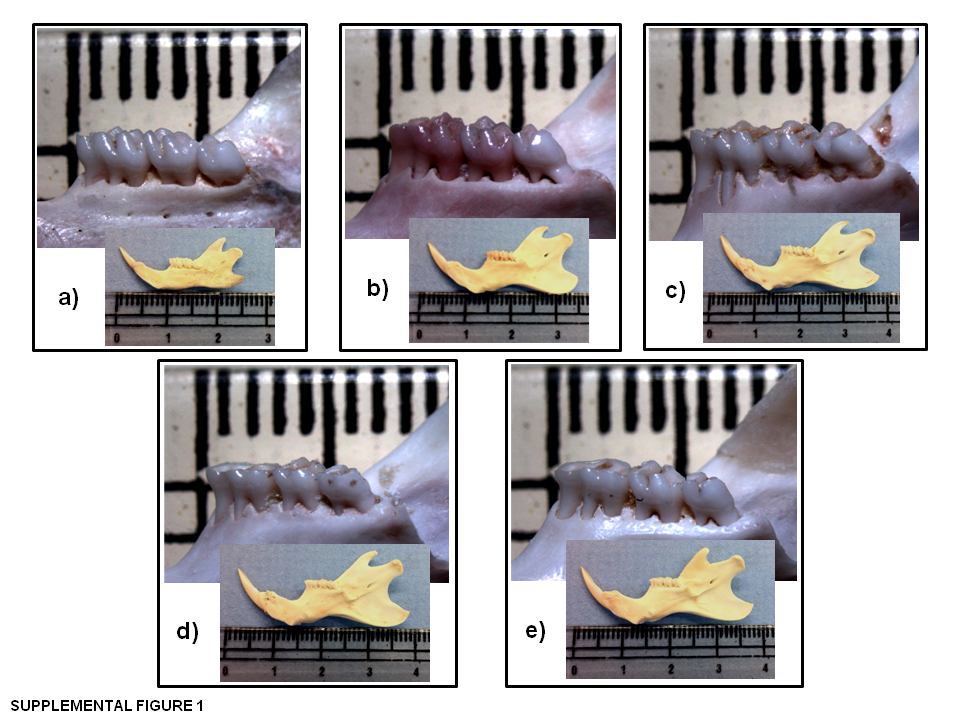

Supplement: Figure S1 — Gross physical changes in hemimandible and molars with age: TOP: Changes in the growing rat molars from younger rats at 1.5, 4, and 6 months. BOTTOM: Changes in rat molars taken from older rats at 10 and 12 months. Both top and bottom rows have insets illustrating respective hemimandibles. Alignment with centimeter ruler shows in the molar view observable increases in occlusal wear and decreases in bone height with age. In the hemimandible inset view, widening of the first molar crown appears to cease at 0.3 cm at 4 months, while mandibles appear to cease at approximately 3.75 cm in length, around 6 months of age. (TIF) [file pone.0035980.s001.tif]

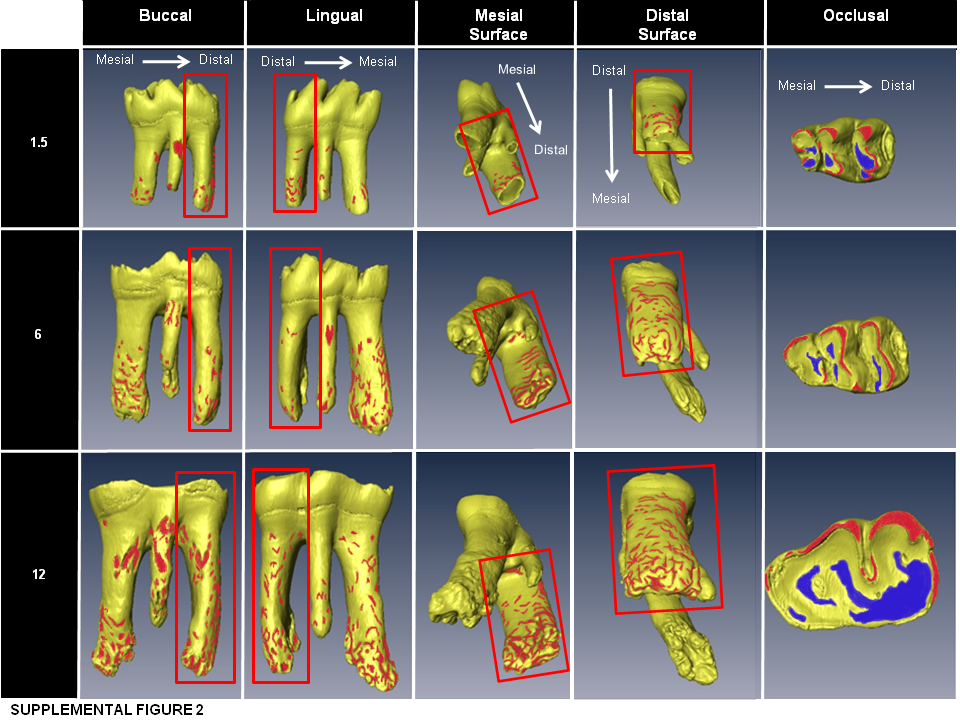

Supplement: Figure S2 — Mesial curving, occlusal wear, and resorption pits with age: MicroXCT reconstructed roots of 1.5, 6, and 12 month first mandibular molars are shown at various buccal, angled, lingual, and occlusal views. Mesial curving becomes prominent at 6 months, as seen in the buccal and angled buccal views. Occlusal wearing is significantly observed with age, especially in the 12 month old molar, as seen in the occlusal view. Buccal and lingual views best demonstrate discrepancies between mesial and distal roots in the first molar. (TIF) [file pone.0035980.s002.tif]

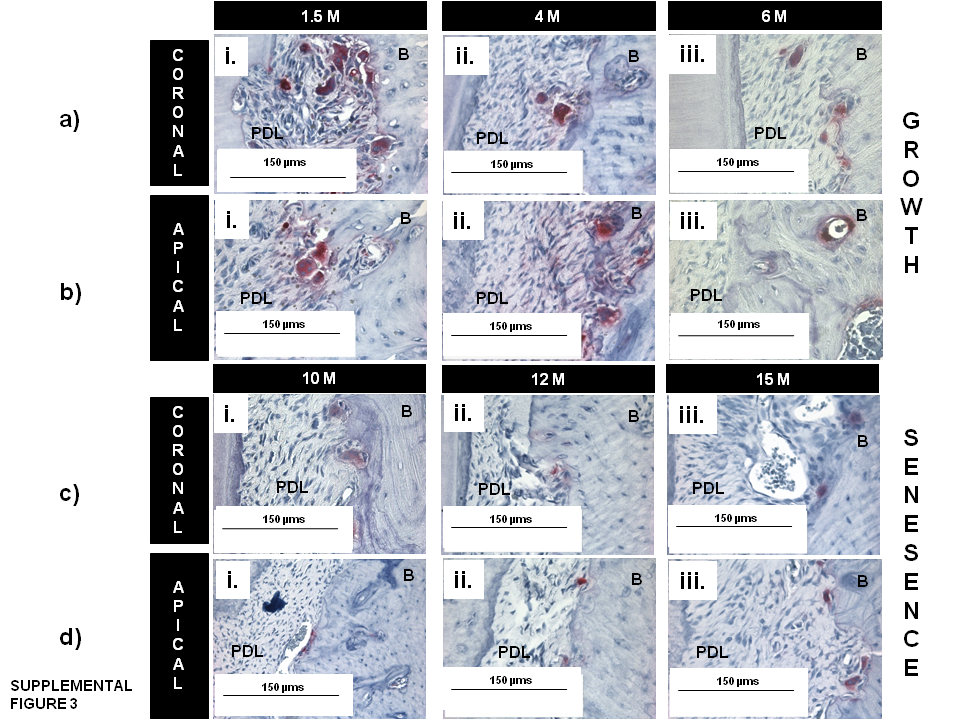

Supplement: Figure S3 — Osteoclastic activity with age: Enlarged at 40X from encircled areas in Figure 1, TRAP localization across all age groups in respective coronal and apical regions is shown. Resorption activity appears to be localized in multinucleated pits as indicated by the red stain surrounding several dark purple nuclei. Osteoclastic activity and multinucleated cells declined with increasing age regardless of region. Scale bar = 150 µm. (TIF) [file pone.0035980.s003.tif]
