# Supplementary material for: Age-Related Adaptation of Bone-PDL-Tooth Complex: Rattus-Norvegicus as a Model System
Source: PLoS One. 2012 Apr 30;7(4):e35980. doi: 10.1371/journal.pone.0035980 (PMC3340399; doi:10.1371/journal.pone.0035980)
Supplement: Appendix S1 — Functional load on rat molars; Distal root of 1st molar; Box-Tidwell Regression Test. (DOCX) [file pone.0035980.s004.docx]

**SUPPORTING INFORMATION**

**Functional load on rat molars; Distal root of 1^st^ molar; Box-Tidwell Regression Test**

**Functional load on rat molars:** Rat pups are gradually weaned and transitioned from a liquid diet to a mixed liquid-hard food and then completely to a hard pellet diet by 1.5 months. Such changes in functional loads (due to stiffness of food ranging from 0.3-0.5 N/mm soft chow to127-158 N/mm hard pellet) over prolonged periods of time likely affect tissue turnover and as a result development [[42](#_ENREF_42)]. Beyond development, the standard hard-pellet rat chow remains a key contributor to the continuous adaptation of the bone-tooth organ in a rat.

**Distal root of 1^st^ molar:** The distal root of the first mandibular molar was consistently chosen to model age-related changes of the rat periodontium because root development and occlusal contact is most complete in young rats (Supplemental Fig. 1). This is in comparison to second and third molars that exhibited hypertrophic growth until 6 months, visible as a widening of the second molar, third molar, and overall hemi-mandible (Supplemental Fig. 1). Biomechanically, the first molar is first to experience compressive forces (occlusal) from functional loading, and lateral forces (mesial-distal forces and/or innate distal drifting) from its neighboring first molar mesial root and second molar distal root. The distal root of the first molar was chosen over the mesial root due to more accurate representation of second and third molars, in shape, size, and angle of roots (Supplemental Fig. 2). For the purposes of this study, the distal root was deemed an appropriate model for observing compounding physiological changes on a functional timeline.

**Box-Tidwell Non-Linear Regression Test**

Averaged Hardness (H_k_) values for secondary cementum (SC) and alveolar bone (AB) from each specimen (n = 4) in all six age groups were calculated. In order to determine if a relationship exists between material hardness and age, we tested for non-linear and linear correlations using the Box-Tidwell Multiple Regression Analysis and Simple Linear Regression Analysis, respectively. To test for non-linearity between hardness values and age, Box-Tidwell was conducted via Statistics Online Computational Resource (SOCR) using a multiple regression model for averaged hardness of cementum and alveolar bone, respectively, in all samples across all age groups (N = 24) with microhardness (GPa) as the dependent variable and age and its natural logarithm (i.e. ln(age)) as independent variables. The microhardness_cementum_ = 0.652 + 0.004*age + 0.0004*(age*ln(age)) + E and microhardness_alveolar bone_ = 0.785 – 0.0026*age + 0.002*(age*ln(age)) + E, (where E is the error term) concluded that age*ln(age) was not significantly different from zero (for cementum P = 0.989 and for alveolar bone P = 0.945). Thus, no significant non-linear relationship exists between age and microhardness. Since there were no significant non-linearities, a simple regression model was plotted to determine linearity between averaged hardness of mineralized tissues in all specimens. The resultant equations: microhardness_cementum_ = 0.650 + 0.005*age and hardness_alveolar bone_ = 0.771 + 0.004*age demonstrated that age was not significantly different from zero (both P-values >0.46), suggesting no significant linear relationship. Since both non-linear and linear relationships between age and microhardness were not evident, there appears to be no significant age effect on hardness.

Additionally, we wanted to determine if there were significant differences between alveolar bone and secondary cementum hardness across all age groups. Analysis of Covariance (ANCOVA) and multiple regression analysis for averaged hardness of secondary cementum and alveolar bone as the dependent variable and age, material, and age*material as the independent variables, were modeled using SOCR. The resultant equation: microhardness = 0.762 + 0.004*age – 0.105*material + E concluded no significant relationship between age and hardness (P= 0.194), supporting our previously mentioned conclusions. However, ANCOVA did demonstrate that alveolar bone was significantly harder than cementum (mean difference = -0.105; 95% confidence interval: -0.163, -0.046; P = 0.007). Interestingly, age*material (P= 0.80) demonstrated no significant relationship between material hardness. Although bone was significantly harder than cementum, the difference does not differ by age. Thus, it appears that the differences between cementum and alveolar bone hardness do not differ by age.
